# *Candida albicans* Adhesins Als1 and Hwp1 Modulate Interactions with *Streptococcus mutans*

**DOI:** 10.3390/microorganisms11061391

**Published:** 2023-05-25

**Authors:** Loyse Martorano-Fernandes, James S. Goodwine, Antônio Pedro Ricomini-Filho, Clarissa J. Nobile, Altair Antoninha Del Bel Cury

**Affiliations:** 1 Department of Prosthodontics and Periodontology, Piracicaba Dental School, University of Campinas (UNICAMP), Piracicaba 13414-903, SP, Brazil; loyse_martorano@hotmail.com; 2 Department of Molecular and Cell Biology, School of Natural Sciences, University of California Merced, Merced, CA 95343, USA; jgoodwi1@gmail.com; 3 Department of Physiological Science, Piracicaba Dental School, University of Campinas (UNICAMP), Piracicaba 13414-903, SP, Brazil; ricomini@unicamp.br; 4 Health Sciences Research Institute, University of California Merced, Merced, CA 95343, USA

**Keywords:** *Candida albicans*, *Streptococcus mutans*, biofilms, polymicrobial biofilms, dual-species biofilms, oral cavity, Als1, Hwp1, interspecies interactions, microbiota

## Abstract

*Candida albicans* and *Streptococcus mutans* are known to synergistically interact with each other in the oral cavity. For example, glucosyltransferase B (GtfB), secreted by *S. mutans*, can bind to the *C. albicans* cell surface, promoting dual-species biofilm formation. However, the fungal factors mediating interactions with *S. mutans* are unknown. The *C. albicans* adhesins Als1, Als3, and Hwp1 are key players in *C. albicans* single-species biofilm formation, but their roles, if any, in interacting with *S. mutans* have not been assessed. Here, we investigated the roles of the *C. albicans* cell wall adhesins Als1, Als3, and Hwp1 on forming dual-species biofilms with *S. mutans*. We assessed the abilities of the *C. albicans* wild-type *als1*Δ/Δ, *als3*Δ/Δ, *als1*Δ/Δ/*als3*Δ/Δ, and *hwp1*Δ/Δ strains to form dual-species biofilms with *S. mutans* by measuring optical density, metabolic activity, cell enumeration, biomass, thickness, and architecture of the biofilms. We observed that the *C. albicans* wild-type strain formed enhanced dual-species biofilms in the presence of *S. mutans* in these different biofilm assays, confirming that *C. albicans* and *S. mutans* synergistically interact in the context of biofilms. Our results reveal that *C. albicans* Als1 and Hwp1 are major players in interacting with *S. mutans*, since dual-species biofilm formation was not enhanced when the *als1*Δ/Δ or *hwp1*Δ/Δ strains were cultured with *S. mutans* in dual-species biofilms. Als3, however, does not seem to play a clear role in interacting with *S. mutans* in dual-species biofilm formation. Overall, our data suggest that the *C. albicans* adhesins Als1 and Hwp1 function to modulate interactions with *S. mutans* and could be potential targets for future therapeutics.

## 1. Introduction

The opportunistic fungal pathogen *Candida albicans* is a normal colonizer of the oral cavity, gastrointestinal tract, and genitourinary tract of healthy humans. However, due to host environmental changes or immunocompromisation, *C. albicans* can become pathogenic and cause superficial and disseminated infections [1,2]. Biofilm formation is a common virulence factor of *C. albicans* that enhances its persistence and pathogenicity in the host [2]. In the oral cavity, *C. albicans* alone can form biofilms and cause infections [1], but *C. albicans* can also form synergistic biofilms with certain bacteria, such as those in the *Streptococcus* genus [3,4]. These interspecies interactions have been shown to enhance microbial colonization [5,6] and persistence, particularly in the oral cavity [7,8]. The presence of *Streptococcus mutans*, for example, is known to lead to the upregulation of common *C. albicans* virulence genes, such as *HWP1*, *SAP4*, and *SAP6*, when *S. mutans* and *C. albicans* are co-cultured together in biofilms [9,10,11]. In addition, these dual-species biofilms have been shown to cause enhanced tissue invasion and damage to the oral epithelium compared to single-species biofilms of *S. mutans* and *C. albicans* alone [9].

Fungal cell wall proteins are important for mediating cell–cell interactions between *C. albicans* cells. For example, the *C. albicans* cell wall adhesins Als1 and Als3 in the agglutinin-like sequence (Als) family are important for *C. albicans* cell–cell interactions during single-species biofilm formation [12,13,14]. In addition, Hwp1, a protein expressed on the *C. albicans* hyphal cell surface, is also important for promoting cell–cell interactions and single-species biofilm formation in *C. albicans* [15,16].

On the bacterial side, *S. mutans* is known to secrete a glucosyltransferase, GtfB, that has been shown to promote coaggregation between *S. mutans* and other microorganisms, including *C. albicans*, enhancing dual-species biofilm formation [7,17,18]. GtfB has been shown to bind to different sites along the *C. albicans* cell well [7,18], suggesting that specific fungal cell wall components could be involved in mediating its binding.

Here, we investigate the roles of the *C. albicans* cell wall proteins Als1, Als3, and Hwp1 on dual-species biofilm formation between *S. mutans* and *C. albicans.* Our results reveal that *C. albicans* Als1 and Hwp1 function to modulate interactions with *S. mutans* in the context of dual-species biofilms.

## 2. Material and Methods

### 2.1. Experimental Design

Experiments followed established protocols designed to evaluate aspects of biofilm formation [19]. Single-species and dual-species biofilms were compared to assess the roles of *C. albicans* Als1, Als3, and Hwp1 proteins in interacting with *S. mutans* using *C. albicans* mutant strains deleted for *ALS1*, *ALS3*, and *HWP1*, respectively. Single-species *C. albicans* wild-type biofilms, single-species *S. mutans* wild-type biofilms, and dual-species *C. albicans-S. mutans* wild-type biofilms were used as controls. Biofilms were formed on the bottoms of 6-well or 96-well plates for 24 h to obtain mature biofilms. Subsequently, biofilms were analyzed to assess optical density, metabolic activity, cell enumeration, biomass, thickness, and architecture. Cell–cell interactions between microorganisms were visualized at the cellular level via optical microscopy. Experiments were performed in triplicate unless stated otherwise.

### 2.2. Strains and Media

All strains used in this study have been previously published and are listed in Table 1. Deletion strains of *C. albicans* lacking *ALS1*, *ALS3*, and *HWP1* as well as a double-deletion strain lacking both *ALS1* and *ALS3* were used for the biofilm assays. In addition, an *S. mutans* GFP-tagged reference strain was used.

*C. albicans* strains were grown from −80 °C glycerol stocks at 30 °C on Yeast Extract Peptone Dextrose (YPD) (Thermo Fisher Scientific, Waltham, MA, USA) agar plates. Overnight *C. albicans* cultures were grown at 30 °C with shaking at 225 rpm in YPD liquid media. *S. mutans* strains were grown from −80 °C glycerol stocks at 37 °C with 10% CO_2_ on Brain Heart Infusion (BHI) (Thermo Fisher Scientific) agar plates. Overnight *S. mutans* cultures were grown on BHI supplemented with 1% glucose (37 °C with 10% CO_2_). RPMI 1640 medium (Sigma Aldrich, St. Louis, MO, USA) was used for the biofilm assays because it supports biofilm formation of both *C. albicans* and *S. mutans* [23].

### 2.3. Biofilm Growth Conditions

Cell enumeration using a hemocytometer relative to the optical density readings was performed for each microorganism to establish a 1:1 ratio of each species in culture, which was equivalent to an OD_600_ of 1 × 10^6^ colony forming units per mL (CFUs/mL) for *C. albicans*, and an OD_600_ of 0.15 × 10^6^ CFUs/mL for *S. mutans*. These CFUs/mL were added to 6-well or 96-well plates for single-species biofilm formation and the same CFUs/mL of each species was added to 6-well or 96-well plates for dual-species biofilm formation. Plates were sealed with Breathe-Easy^®^ sealing membranes and incubated at 37 °C for 90 min at 250 rpm shaking with 10% CO_2_. Cells were washed with phosphate-buffered saline (PBS) to remove non-adhered cells and a fresh RPMI 1640 medium was added to each well. Biofilms were grown for 24 h.

### 2.4. Standard Optical Density Biofilm Assay

The standard biofilm optical density biofilm assay was performed as previously described [19]. Following biofilm growth on 96-well plates, the media were aspirated from the wells, and the biofilm formed on the bottom of each well was measured according to OD_600_ readings obtained using a plate reader. An average of 24 readings per well were obtained and normalized by subtracting the OD_600_ reading of a blank well containing an RPMI 1640 medium only.

### 2.5. Cell Metabolism Biofilm Assay

The 2,3-bis-(2-methoxy-4-nitro-5-sulfophenyl)-2H-tetrazolium-5-carboxanilide (XTT) reduction assay was performed to measure the biofilm metabolic activity, as previously described [19]. A mixture of 0.5 mg/mL of XTT (Sigma Aldrich) in PBS and 0.32 mg/mL of phenazine methosulfate (Sigma Aldrich) in water was added to each well and incubated in the dark for 30 min. After incubation, OD_492_ readings were taken in a plate reader.

### 2.6. Biofilm Cell Enumeration Assay

The biofilm cell enumeration assay was performed as previously described [19]. After the 24 h period of biofilm formation, the media were aspirated from the 96-well plates, and the biofilms were washed to remove non-adhered cells. The biofilm formed on the bottom of each well was vigorously scraped using a pipette tip and resuspended in PBS. Cell suspensions were homogenized and serially diluted in PBS. Aliquots were plated onto YPD plates for *C. albicans* (grown at 30 °C for 48 h) and onto Mitis Salivarius Agar (MSA) plates for *S. mutans* (grown at 37 °C for 72 h) to enumerate colony-forming units (CFUs) for each species. We note that although *C. albicans* colonies can also grow on MSA plates, they are clearly distinct from the *S. mutans* colonies, and supplementation of the medium with antibiotics is not necessary. Additionally, with MSA plates, under these growth conditions, *S. mutans* colonies come up first and are easily counted before *C. albicans* colonies begin to appear on the plates.

### 2.7. Biofilm Biomass Determination

The biofilm biomass assay (i.e., dry weight assay) was performed as previously described [19]. Biofilms were grown on the bottoms of 6-well plates. Media was aspirated from the wells and PBS was added to each well. Biofilms were vigorously scraped and resuspended from the bottoms of each well using a pipette tip. Biofilm cell suspensions were aspirated and filtered onto mixed cellulose esters membranes (Millipore, Burlington, MA, USA) using a filtration device (Millipore). Membranes were subsequently dried for 24 h at 37 °C, and the weights of the membranes (in mg) were measured to obtain the dry weights of the biofilms. Data were normalized by subtracting the average weight of the control (media only) membrane.

### 2.8. Confocal Scanning Laser Microscopy (CSLM) Biofilm Assay

The confocal scanning laser microscopy (CSLM) biofilm assay was performed as previously described [19], with slight modifications (detailed below). Representative images of biofilms (n = 3 per group) were obtained via CSLM using a Zeiss LSM 880 upright confocal microscope. Biofilms were grown on the bottoms of silicone squares for 24 h using the same biofilm growth conditions described above. Biofilms were fixed with a formaldehyde solution (38% formaldehyde in water). *C. albicans* biofilms were stained with Concanavalin A-Alexa Fluor 594 (Sigma Aldrich) and visualized using a 555 nm diode (red) laser. The *S. mutans* GFP-tagged strain was visualized via excitation at 488 nm (green). Z-Stacks were obtained at 652 × 652 pixels, imaging every 0.5 μm intervals using a water-dipping 40X objective lens. The .czi files were analyzed using the project stacks function in ImageJ to generate side views. Biofilm thickness was measured in µm by taking the median thicknesses of each strain/condition (n = 3) using the Zeiss ZEN software version 3.6. Three .czi files of each sample (containing a combined total of at least 100 cells in a 0.5 μm interval) were used to quantify the average number of potential physical interactions between *C. albicans* and *S. mutans* cells.

### 2.9. Optical Microscopy

Planktonic cultures of *C. albicans* and *S. mutans* were grown separately overnight in YPD (for *C. albicans*), and BHI supplemented with 1% glucose (for *S. mutans*). Cultures were diluted to an OD_600_ of 0.5 in an RPMI 1640 medium and co-cultured at 37 °C with 10% CO_2_ for 4 h, with shaking at 250 rpm. An aliquot of the co-cultures was visualized using an EVOS FL microscope with a 60× oil immersion objective. Representative images of potential physical interactions were obtained for three independent experiments. Three images of each experiment (containing a combined total of at least 100 cells) were used to quantify the average number of potential physical interactions between *C. albicans* and *S. mutans* cells.

### 2.10. Statistical Analyses

Data were analyzed using the Statistical Package for the Social Sciences (SPSS) software (version 22.0). Means and standard deviations were calculated. Based on the results of Levene’s test for equality of variances, Student’s unpaired two-tailed *t*-tests for unequal variance or one-way ANOVAs were performed. GraphPad Prism software (version 9.4) was used to generate the graphs.

## 3. Results

**Figure 1:**
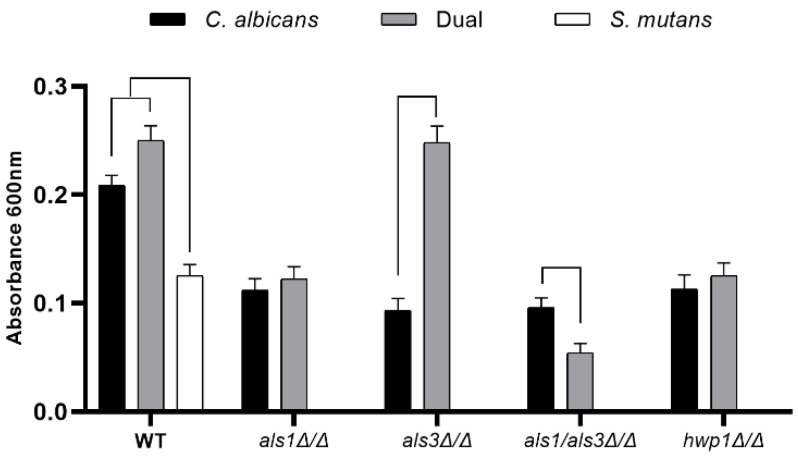
Standard optical density biofilm assay of single-species and dual-species biofilms. Data shown include n = 6 per group. A line connecting two bars indicates *p* < 0.05 as determined using Student’s unpaired two-tailed t-tests assuming unequal variance. Error bars represent standard deviations.

To test our hypothesis that Als1, Als3, and Hwp1 play roles in dual-species *C. albicans*-*S. mutans* biofilm formation, we first compared the biofilms formed by the single-species and dual-species biofilms of each strain using the standard optical density biofilm assay [19], which correlates with biofilm thickness. Using this assay, we found a synergistic interaction between *C. albicans* and *S. mutans*, where an increase in optical density was observed for the dual-species wild-type biofilms, compared to the single-species *C. albicans* wild-type biofilms and the single-species *S. mutans* wild-type biofilms (Figure 1). We found that the presence of *S. mutans* had no effect on the biofilm formation capacity of the *C. albicans als1*Δ/Δ or *hwp1*Δ/Δ strains compared to the single-species *als1*Δ/Δ or *hwp1*Δ/Δ strains, respectively; however, the presence of *S. mutans* strikingly increased the biofilm formation capacity of the *C. albicans als3*Δ/Δ strain compared to the single-species *als3*Δ/Δ strain (Figure 1). We note that complementation (addback) strains *als1*Δ/Δ + *ALS1*, *hwp1*Δ/Δ + *HWP1*, and *als3*Δ/Δ + *ALS3* restored the mutant phenotypes for dual-species biofilms back to near wild-type levels (*als1*Δ/Δ + *ALS1*, OD_600_ = 0.18 ± 0.01; *hwp1*Δ/Δ + *HWP1*, OD_600_ = 0.16 ± 0.02; *als3*Δ/Δ + *ALS3*, OD_600_ = 0.15 ± 0.02). Interestingly, the presence of *S. mutans* impaired the biofilm formation capacity of the *C. albicans als1*Δ/Δ*/als3*Δ/Δ double-deletion strain compared to the single-species *als1*Δ/Δ*/als3*Δ/Δ strain (Figure 1).

**Figure 2:**
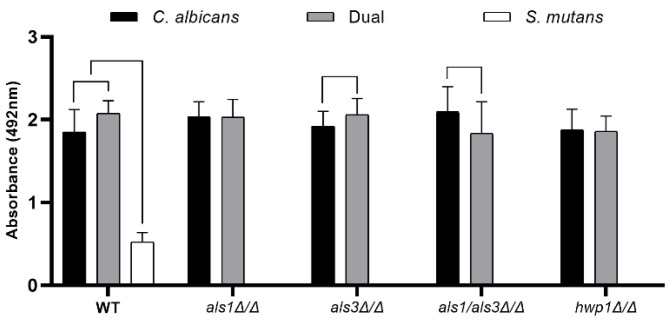
XTT metabolic activity assay of single-species and dual-species biofilms. Data shown include n = 6 per group. A line connecting two bars indicates *p* < 0.05 as determined using Student’s unpaired two-tailed *t*-tests assuming unequal variance. Error bars represent standard deviations.

Similar results to the optical density biofilm assay were obtained for the XTT cell metabolism biofilm assay and the dry weight biofilm biomass assay [19], where an increase in metabolic activity (Figure 2) and biomass (Table 2) was observed for the dual-species wild-type biofilms, compared to the single-species *C. albicans* wild-type biofilms and the single-species *S. mutans* wild-type biofilms. In addition, the presence of *S. mutans* increased the metabolic activity (Figure 2) and biomass (Table 2) of the *C. albicans als3*Δ/Δ strain compared to the single-species *als3*Δ/Δ strain, but had no effect on the metabolic activity (Figure 2) or biomass (Table 2) of the *C. albicans als1*Δ/Δ or *hwp1*Δ/Δ strains compared to the single-species *als1*Δ/Δ or *hwp1*Δ/Δ strains, respectively. Likewise, the presence of *S. mutans* reduced the metabolic activity (Figure 2) and biomass (Table 2) of the *C. albicans als1*Δ/Δ*/als3*Δ/Δ double-deletion strain compared to the single-species *als1*Δ/Δ*/als3*Δ/Δ strain.

**Figure 3:**
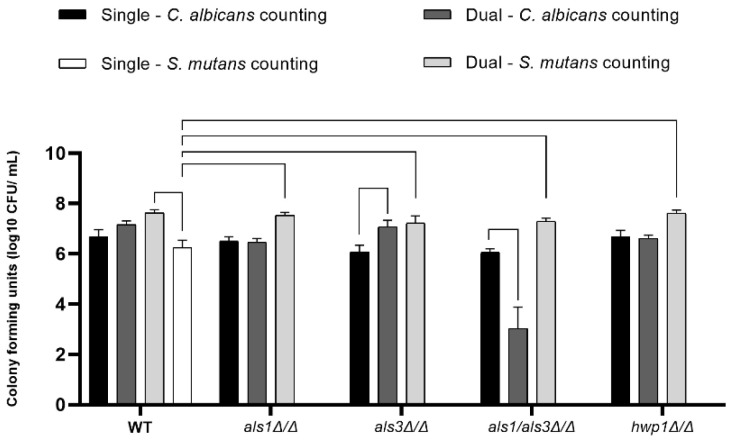
Cell enumeration in biofilms. Data shown are CFUs on a log_10_ scale (n = 4 per group). A line connecting two bars indicates *p* < 0.05 according to one-way ANOVA tests. Error bars represent standard deviations.

To determine the number of cells of each species present in the biofilms, we next measured the CFUs for each biofilm sample [19]. Overall, *S. mutans* CFUs were higher in all dual-species biofilms compared to *S. mutans* CFUs in all single-species biofilms (Figure 3), indicating that *S. mutans* benefits from *C. albicans* by increasing its cell population. Furthermore, *C. albicans* CFUs were lower in dual-species biofilms of the *C. albicans als1*Δ/Δ*/als3*Δ/Δ double-deletion strain compared to the single-species *als1*Δ/Δ*/als3*Δ/Δ strain (Figure 3). *C. albicans* CFUs were also lower in the single-species biofilms of the *als3*Δ/Δ strain compared to the dual-species biofilms of the *als3*Δ/Δ strain (Figure 3).

**Figure 4:**
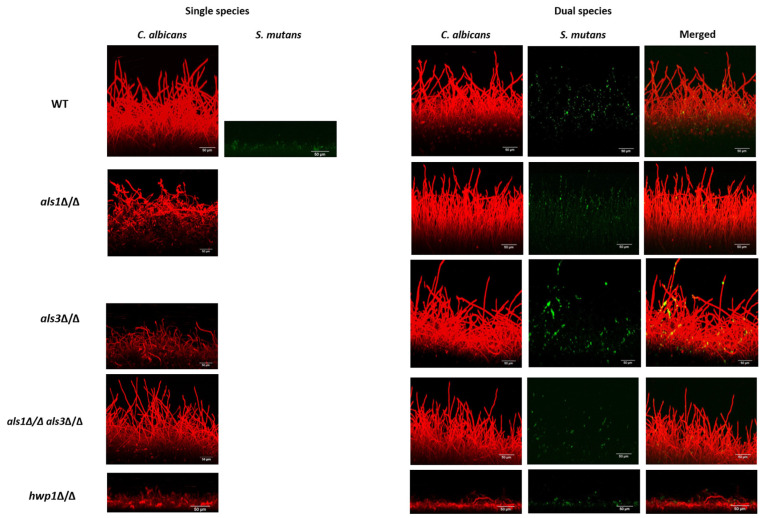
CSLM biofilm assay. Biofilms were imaged using a Zeiss LSM 880 upright confocal microscope. *C. albicans* biofilms were stained with Concanavalin A-Alexa Fluor 594 (red) and visualized using a 555-nm diode laser. The *S. mutans* GFP strain (green) was detected via excitation at 488 nm. Representative images are shown. All Z-Stacks were obtained at 652 × 652 pixels, with imaging undertaken at 0.5 μm intervals using a water-dipping 40X objective. The .czi files were analyzed using the project stacks function in ImageJ to generate side-views. Scales bars = 50 μm.

To assess biofilm architecture and thickness, we next performed confocal scanning laser microscopy (CSLM) assays on the biofilms [19]. Thickness measurements of biofilms were determined based on the CSLM medians of all images taken for a given strain and condition and are reported in Table 3. We observed that wild-type dual-species biofilms were overall thicker than *C. albicans* single-species biofilms as measured in the CSLM side-view images (Figure 4; Table 3). In addition, as expected, all single-species *C. albicans* mutant strains produced defective biofilms (Figure 4; Table 3). Consistent with our results reported above for the other biofilm assays, the thicknesses of the biofilms of the *als1*Δ/Δ and *hwp1*Δ/Δ strains were similar between the single-species and dual-species biofilms (Table 3). In addition, the presence of *S. mutans* in the dual-species biofilms increased the biofilm thickness of the *C. albicans als3*Δ/Δ strain compared to the single-species *als3*Δ/Δ strain (Table 3). Overall, the CSLM results indicate that *S. mutans* benefits from the presence of *C. albicans* in all dual-species biofilms. We note that single-species *S. mutans* biofilms formed thin cell aggregates restricted to the bottoms of the substrates, while in dual-species, the *S. mutans* cells were observed throughout the biofilms, often appearing along *C. albicans* hyphal cells. Specifically, the CSLM images of the dual-species biofilms showed predominance of *S. mutans* cells in close physical proximity to *C. albicans*-elongated hyphal cells in the wild-type *als1*Δ/Δ, *als3*Δ/Δ, and *als1*Δ/Δ*/als3*Δ/Δ strains (Figure 4; Table 4). In dual-species biofilms with the *hwp1*Δ/Δ strain, however, there appeared to be a predominance of *S mutans* cells in close physical proximity to *C. albicans* around yeast-form cells (Figure 4; Table 4).

**Figure 5:**
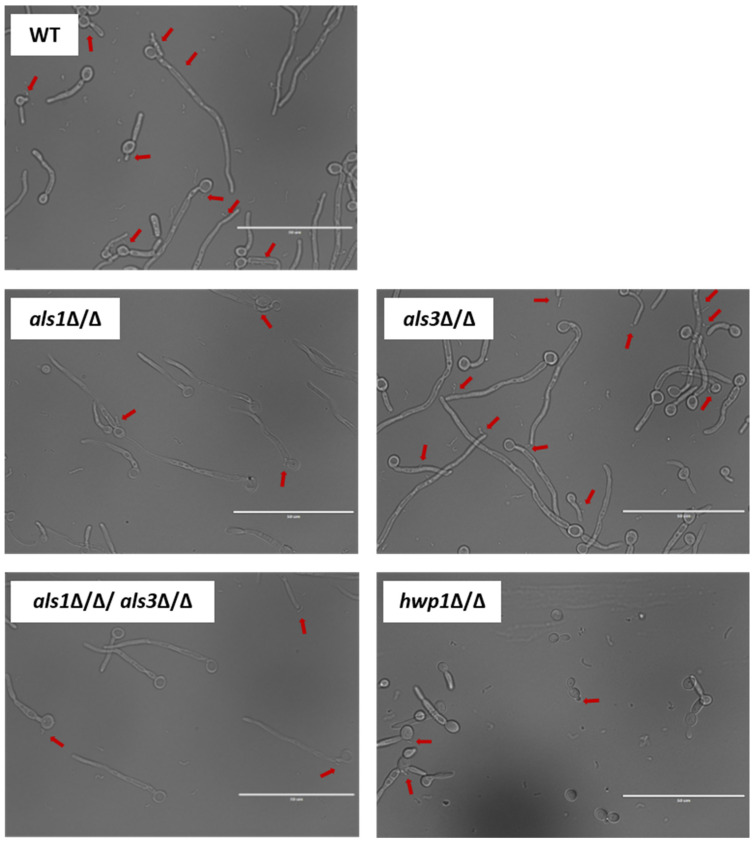
Optical microscopy cellular images of planktonic co-cultures of *C. albicans* and *S. mutans.* Representative images are shown that were taken using an EVOS FL microscope with a 60X oil immersion objective. Red arrows indicate potential binding events of *S. mutans* along *C. albicans* cells. Scales bars = 50 μm.

Given our observation that *S. mutans* cells appeared generally to be in close physical proximity to *C. albicans* hyphal cells in the context of dual-species biofilms (Figure 4; Table 4), we also performed optical microscopy of planktonic cultures to visualize in more detail the physical proximity and/or potential cellular interactions occurring between *C. albicans* and *S. mutans* under non-biofilm co-culture conditions. Co-cultures of the wild-type *C. albicans* and *S. mutans* strains showed on average increased physical proximity of *S. mutans* cells to *C. albicans* hyphal cells compared to yeast-form cells (Figure 5; Table 5). Interestingly, co-cultures of the *C. albicans als1*Δ/Δ, *hwp1*Δ/Δ, and *als1*Δ/Δ*/als3*Δ/Δ strains with *S. mutans* showed strikingly fewer potential binding events on average compared to co-cultures of the *C. albicans* wild-type strain with *S. mutans* (Figure 5; Table 5). Co-cultures of the *C. albicans als3*Δ/Δ strain with *S. mutans* showed similar potential binding events of *S. mutans*, specifically to *C. albicans* hyphal cells, compared to co-cultures of the *C. albicans* wild-type strain with *S. mutans* (Figure 5; Table 5).

## 4. Discussion

The oral cavity is home to thousands of microorganisms existing in biofilm microbial communities and is an important niche in the study of interspecies interactions [24,25]. The opportunistic fungal pathogen *C. albicans* and the opportunistic bacterial pathogen *S. mutans* are prevalent microorganisms in the oral cavity [1,26], where they are known to benefit from one another’s presence [7,8]. When *C. albicans* and *S. mutans* are grown together in biofilms under oral cavity conditions, the presence of *S. mutans* has been shown to enhance fungal virulence by increasing the yeast–hyphal transition in *C. albicans* [8,9]. In addition, under oral cavity conditions, *C. albicans* has been shown to acidify its environment, thereby supporting the growth and metabolism of *S. mutans* [27]. Together, *C. albicans* and *S. mutans* can form pathogenic dual-species biofilms in the oral cavity that can lead to the development of caries and other oral diseases [9].

Cell–cell interactions between *C. albicans* and *S. mutans* are thought to occur through physical binding events between proteins that are either already present on the cell surface and/or that are secreted by one microorganism to mediate binding to the other microorganism [28,29]. For example, glucosyltransferase B (GtfB), secreted by *S. mutans*, can bind to the *C. albicans* cell surface, promoting dual-species biofilm formation between the two species [17]. However, the *C. albicans* factors mediating interactions with *S. mutans* are unknown. Here, we investigated the roles of the fungal cell surface adhesin proteins Als1, Als3, and Hwp1 in interacting with *S. mutans*.

Als1 is a GPI-anchored adhesin that is involved in *C. albicans* single-species biofilm formation, cell–cell and cell-surface interactions, as well as interactions with the host epithelium [14,15,30]. Als1 is known to be required for physically interacting with the oral bacterium *Streptococcus gordonii* [31], and thus it is feasible that Als1 could be a key player in mediating interactions with other oral bacterial species, such as *S. mutans*. Als3 is a GPI-anchored adhesin with 88% amino acid sequence similarity to Als1 [32] that is involved in *C. albicans* single-species biofilm formation and is known to play functionally redundant roles with Als1 [13,15]. Given its functional redundancy with Als1, we reasoned that Als3 could also be a key player in mediating interactions with *S. mutans*. Hwp1 is a well-known *C. albicans* adhesin that is expressed only on hyphal cells and is covalently linked to the fungal cell wall via a remnant of its GPI anchor [33,34]. Like Als1 and Als3, Hwp1 is also important for *C. albicans* single-species biofilm formation [16,35], and we reasoned that it could also be a candidate cell-surface protein for mediating interactions with *S. mutans*. Based on this information, we hypothesized that Als1, Als3, and Hwp1 play roles in interacting with *S. mutans* during dual-species biofilm formation.

To test our hypothesis, we assessed the abilities of the *C. albicans* wild-type *als1*Δ/Δ, *als3*Δ/Δ, *als1*Δ/Δ/*als3*Δ/Δ, and *hwp1*Δ/Δ strains to form dual-species biofilms with *S. mutans* by measuring the optical density, metabolic activity, cell enumeration, biomass, thickness, and architecture of the biofilms. We also performed confocal and optical microscopy assays under biofilm and planktonic conditions to quantify potential binding interactions occurring between *C. albicans* and *S. mutans* cells, where we observed that *S. mutans* cells were generally found to be in close proximity with *C. albicans* hyphal cells rather than yeast-form cells. We observed that the *C. albicans* wild-type strain formed enhanced dual-species biofilms in the presence of *S. mutans* in all of the different biofilm assays, confirming that *C. albicans* and *S. mutans* synergistically interact in the context of dual-species biofilms. Our results revealed that *C. albicans* Als1 and Hwp1 are major players in interacting with *S. mutans* since dual-species biofilm formation was not enhanced when the *C. albicans als1*Δ/Δ or *hwp1*Δ/Δ strains were cultured with *S. mutans* in dual-species biofilms. The role of Als3, however, in interacting with *S. mutans* is not as straightforward. In general, we observed that when the *C. albicans als3*Δ/Δ strain was cultured with *S. mutans* in dual-species biofilms, biofilm formation was rescued to varying degrees depending on the biofilm assay used. In all of the assays, the rescue was within or close to wild-type levels, indicating that Als3 does not seem to play a clear role in interacting with *S. mutans* in dual-species biofilms. Finally, in all of the assays used, the combined absence of Als1 and Als3 in the *als1*Δ/Δ*/als3*Δ/Δ strain showed a detrimental impact on dual-species biofilm formation beyond that of either of the single *als1*Δ/Δ or *als3*Δ/Δ strains alone. Although there is not a clear-cut explanation for this finding, one possibility is that when both *C. albicans* Als1 and Als3 are absent, and with the additional presence of *S. mutans* cells, the overall structural integrity of the dual-species biofilm is disrupted. We note that our dual-species biofilm experiments were performed using a 1:1 ratio of *S. mutans*-to-*C. albicans* cells to seed the biofilms. We chose this ratio so that each species could be equally represented irrespective of their cell size differences and because we obtained robust dual-species biofilms under these seeding conditions. Nonetheless, it is possible that the dual-species biofilm architectures and interactions that we observed here could be population-dependent and would change if different seeding ratios were used.

Given our findings that *C. albicans* Als1 and Hwp1 function to modulate interactions with *S. mutans*, and that these two proteins are located on the *C. albicans* cell surface, Als1 and Hwp1 could be promising therapeutic targets to consider in the future development of novel therapeutics to treat polymicrobial biofilm infections.

## Figures and Tables

**Table 1:** Strains used in this study.

Strain	Source
*C. albicans* wild-type (WT) SC5314	[20]
*S. mutans* UA 159	[21]
*S. mutans* pDL278_P23-sfgfp	[22]
*C. albicans als1*Δ/Δ	[15]
*C. albicans als3*Δ/Δ	[15]
*C. albicans als1*/*als3*Δ/Δ	[13]
*C. albicans hwp1*Δ/Δ	[16]
*C. albicans als1*Δ/Δ + *ALS1*	[13]
*C. albicans als3*Δ/Δ + *ALS3*	[15]
*C. albicans hwp1*Δ/Δ + *HWP1*	[16]

**Table 2:** Biofilm dry weights.

Strain	Dry Weight (Mean ± SD) (mg)	
	Single	Dual
*C. albicans* WT	10.09 ± 0.26	12.03 ± 0.54 *
*S. mutans* UA159	1.88 ± 0.28	-
*C. albicans als1*Δ/Δ	4.75 ± 0.22	5.16 ± 0.27
*C. albicans als3*Δ/Δ	4.56 ± 0.23	8.06 ± 0.27 *
*C. albicans als1/als3*Δ/Δ	4.43 ± 0.32	3.31 ± 0.27 *
*C. albicans hwp1*Δ/Δ	3.38 ± 0.20	3.74 ± 0.24

* Statistically significant difference (*p* < 0.05) between single-species and dual-species biofilms according to Student’s unpaired two-tailed t-tests assuming unequal variance.

**Table 3:** Median biofilm thicknesses from CSLM images.

Strain	Thickness (n = 3) (µm) ± SD	
	Single	Dual
*C. albicans* WT	235 ± 10	265 ± 10
*C. albicans als1*Δ/Δ	100 + 15	100 ± 10
*C. albicans als3*Δ/Δ	55 ± 5	130 ± 10
*C. albicans als1/als3*Δ/Δ	90 ± 10	70 ± 10
*C. albicans hwp1*Δ/Δ	15 ± 5	15 ± 5

**Table 4:** Average number of potential binding interactions observed using CSLM between *C. albicans* and *S. mutans* cells under dual-species biofilm conditions.

CO-Culture	*C. albicans* Morphology (n = 3) (%) *	
	Yeast	Hyphae
*C. albicans* WT + *S. mutans* WT	5 ± 2	52 ± 12
*C. albicans als1*Δ/Δ + *S. mutans* WT	2 ± 1	50 ± 9
*C. albicans als3*Δ/Δ + *S. mutans* WT	7± 4	45 ± 9
*C. albicans als1/als3*Δ/Δ + *S. mutans* WT	5 ± 1	31 ± 8
*C. albicans hwp1*Δ/Δ + *S. mutans* WT	14 ± 5	6 ± 3

* The hyphae category consists of both hyphae and pseudohyphae morphologies.

**Table 5:** Average number of potential binding interactions observed via optical microscopy between *C. albicans* and *S. mutans* cells under planktonic conditions.

Co-Culture	*C. albicans* Morphology (n = 3) (%) *	
	Yeast	Hyphae
*C. albicans* WT + *S. mutans* WT	0 ± 1	18 ± 4
*C. albicans als1*Δ/Δ + *S. mutans* WT	0 ± 0	3 ± 2
*C. albicans als3*Δ/Δ + *S. mutans* WT	0 ± 0	22 ± 6
*C. albicans als1/als3*Δ/Δ + *S. mutans* WT	0 ± 0	4 ± 2
*C. albicans hwp1*Δ/Δ + *S. mutans* WT	1 ± 1	2 ± 1

* The hyphae category consists of both hyphae and pseudohyphae morphologies.

## Data Availability

Data is contained within the article.
